# Production and proteomic characterisation of purified protein derivative from *Mycobacterium avium *subsp. *paratuberculosis*

**DOI:** 10.1186/1477-5956-10-22

**Published:** 2012-03-26

**Authors:** James W Wynne, Brian J Shiell, Michelle L Colgrave, Jill A Vaughan, Gary Beddome, Wojtek P Michalski

**Affiliations:** 1 Australian Animal Health Laboratory, CSIRO Livestock Industries, Geelong, Victoria 3219, Australia; 2 Queensland Bioscience Precinct, CSIRO Livestock Industries, St Lucia, Queensland 4067, Australia

**Keywords:** Johne's disease, Johnin PPD, PPDj, Interferon gamma, Diagnostic antigen, Mass spectrometry

## Abstract

**Background:**

Effective diagnosis of Johne's disease (JD), particularly at the stage of early subclinical infection, remains one of the greatest challenges for the control of JD worldwide. The IFN-γ test of cell mediated immunity is currently one of the most suitable diagnostics for subclinical infections, however a major limitation of this test is the lack of a standardised purified protein derivative (PPD) antigen (also referred to as Johnin PPD or PPDj). While attempting to replace PPDj with more specific individual antigens is an attractive proposition, bacterial culture derived PPDj remains the most effective antigen preparation for the diagnosis of subclinical JD. It may be possible to increase the reproducibility and specificity of PPDj preparations by further characterising and standardising the PPDj production.

**Results:**

Using a standardised protocol, five in-house preparations of PPDj were prepared from cultures of *Mycobacterium avium *subsp. *paratuberculosis *(MAP). Compared to PPDs obtained from other institutes/laboratories, these preparations appeared to perform similarly well in the IFN-γ test. Although the broad proteomic composition of all PPDj preparations was remarkably similar, the absolute abundance of individual proteins varied markedly between preparations. All PPDj preparations contained common immunogenic proteins which were also observed in PPD preparations from *Mycobacterium avium *subsp. *avium *(PPDa) and *Mycobacterium bovis *(PPDb). Temporal difference in protein secretion of *in vitro *cultured MAP was observed between 20 and 34 weeks suggesting that the age of MAP culture used for PPDj preparations may markedly influence PPDj composition.

**Conclusions:**

This study describes a protocol for the production of PPDj and its subsequent proteomic characterisation. The broad proteomic composition of different preparations of PPDj was, for the most part, highly similar. Compositional differences between PPDj preparations were found to be a direct reflection of genetic differences between the MAP strain types used to produce these preparations and the age of MAP cultures they were derived from. A number of conserved immunogenic proteins, such as members of the cutinase-like protein family, were found to be more abundant in PPDj compared to PPDa and should be considered as possible diagnostic antigens for the future.

## Background

Johne's disease (JD) is a significant animal health issue worldwide. JD is a chronic infectious enteritis of wild and domestic ruminants. Caused by the Gram positive, acid fast bacilli, *Mycobacterium avium *subsp. *paratuberculosis *(MAP), JD is responsible for significant financial losses to many livestock industries, most notably dairy and beef cattle production [1]. Asymptomatic subclinically infected animals pose a significant challenge for the control and management of JD. From the early stages of infection animals have the ability to excrete large quantities of viable bacilli into the environment which can subsequently reinfect other members of the herd. The accurate diagnosis and eradication of subclinically infected animals is therefore vital for control and management of JD worldwide. Although not proven, MAP may also act as a zoonotic agent in some human diseases [2-4].

A range of diagnostic tests, varying in their sensitivity, specificity and suitability have been trialled for the diagnosis of MAP infections [5-9]. Serological-based tests such as ELISA [9], gel immuno-diffusion [7,10] and complement fixation [5] tests have proven successful for the diagnosis of the clinical stages of disease when a robust antibody response has developed. Serological-based tests, however, have failed to diagnose both early and subclinical stages of infection. Traditionally, the early and subclinical stages of infection were thought to be dominated by cell-mediated immunity (CMI) rather than circulating antibodies [11]. Recent evidence however suggests that JD infected sheep are more likely to have a combined CMI and antibody response during the early stages of infection [12]. The CMI response against MAP is characterised by a Th1-type cellular response leading to the secretion of a number of cytokines, including interferon gamma (IFN-γ) [11].

Diagnostic tests based on measuring CMI response have proven to be more suitable for the detection of subclinical stages of infection compared to serological-based tests [5]. The most common test of CMI for JD diagnosis relies on the quantification of released IFN-γ following antigenic stimulation of peripheral blood lymphocytes. Originally developed for the diagnosis of bovine tuberculosis (BOVIGAM™) [13], this IFN-γ test has also been adapted for the diagnosis of subclinical MAP infections across a range of species including cattle [14,15], sheep [5] and goats [8]. This test is based on an ELISA and relies on the use of purified protein derivative (PPD) as the stimulating antigen. Johnin PPD (PPDj) is a crude preparation of MAP culture inactivated by heat treatment, precipitated with trichloroacetic acid (TCA) and resuspended in phosphate buffer containing glucose and phenol.

While described in the Australia and New Zealand Standard Diagnostic Procedure (ANZSDP; http://www.scahls.org.au) for JD diagnosis, the IFN-γ test is not routinely used in Australia due to its low specificity to diagnose JD over non-MAP mycobacterial exposure. Indeed, several studies have demonstrated JD infected animals cross react with PPD antigen preparations from *Mycobacterium bovis *(PPDb) and *Mycobacterium avium *(PPDa) [6,15]. Such cross reactivity is most likely due to the presence of common immunogenic proteins conserved across the *Mycobacterium *genus. Many of these proteins are major components of PPDb and PPDa [16,17]. Increasing specificity through, either the replacement of PPDj with individual antigens, or refining PPD composition, may greatly improve this diagnostic. PPDj may be refined by simply standardising its production, including using a common MAP reference strain, an optimised harvest time point and production procedure.

Another major drawback of the IFN-γ test, which contributes to its low specificity, concerns the lack of standardised PPDj preparations. While PPDj is now produced by a number of different institutes/laboratories, its potency to induce an IFN-γ response differs significantly between preparations [7,10,18]. Consequently the concentration of PPDj antigen used in IFN-γ tests varies considerably between laboratories [15]. Globally, a range of genetically diverse MAP strains have been employed to prepare PPDj [19]. In Canada alone as many as six different MAP strains have been used, some of which have been cultured within the laboratory for many generations [19]. The different MAP strains used to prepare PPDj may significantly contribute to the variability between PPDj preparations. Indeed, strain differences have been shown to significantly alter protein expression profiles. A comparison between the laboratory-adapted reference isolate K10 with a wild type bovine isolate 187 demonstrated the latter has increased expression of a number of proteins compared to K10 [20]. Different MAP strains also demonstrate variation in growth dynamics which may significantly affect the composition of PPDj depending on the harvest time point [21].

The variable non-standardised nature of PPDj makes comparisons between different laboratories difficult. While attempts to replace PPDj with individual antigens have proven encouraging [22], PPDj remains the most widely used diagnostic antigen for subclinical JD diagnosis using the IFN-γ assay. We believe that by careful standardisation and characterisation of PPDj it may be possible to improve this diagnostic antigen and also, in the process, identify new antigen targets. To this end, our laboratory has produced PPDj in-house over the past decade following a standardised laboratory protocol. These PPDj preparations have been utilised in a number of published studies and have performed as well as other available PPDj preparations in their ability to diagnose MAP infections [23-25].

In this study we describe a standardised protocol for the production of PPDj within our laboratory. The proteomic composition of these preparations were then characterised by mass spectrometry and compared to a selection of PPD preparations sourced from other institutes/laboratories. Temporal changes in protein secretion of *in vitro *cultured MAP were also investigated in an attempt to identify the optimal harvest time point for PPDj preparation. Throughout this process a number of potential antigens were identified and these may serve as possible diagnostic markers for JD infection. Such antigens may even offer an alternative to the PPDj in the future.

## Results and Discussion

**Figure 1:**
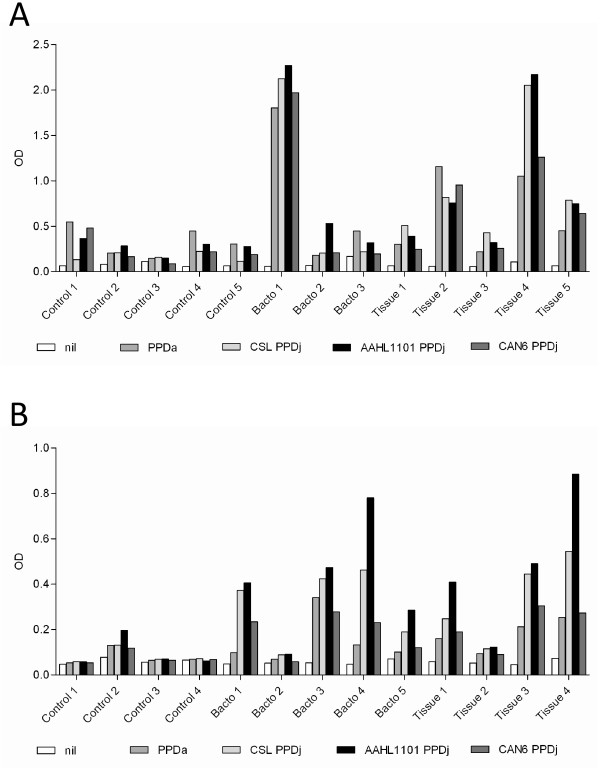
**Results of IFN-γ assay in Holstein-Friesian cattle (A) and Merino sheep (B)**. Control, denotes control (non-MAP infected) animals. 'Bacto', denotes those animals infected with *in vitro *cultured MAP. 'Tissue', denotes animals infected via consumption of a tissue biopsy from a JD positive donor. The individual animal number is also indicated following the group description.

The ability of the in-house produced AAHL1101 PPDj to generate an IFN-γ response was demonstrated across 13 Holstein-Friesian cattle and 13 Merino sheep experimentally infected with MAP (Figure 1A and 1B). The IFN-γ response of infected cattle against AAHL1101 PPDj was not significantly different compared to the CSL or CAN6 PPDj preparations (*p *> 0.05). Interestingly, the IFN-γ response of infected sheep against AAHL1101 PPDj was significantly greater compared to the CAN6 PPDj preparation (*p *< 0.05), but not the CSL preparation (*p *> 0.05). Infected cattle also responded against PPDa. In one instance this response was greater than against PPDj. This result highlights the cross-reactivity inherent with PPDa and PPDj.

**Figure 2:**
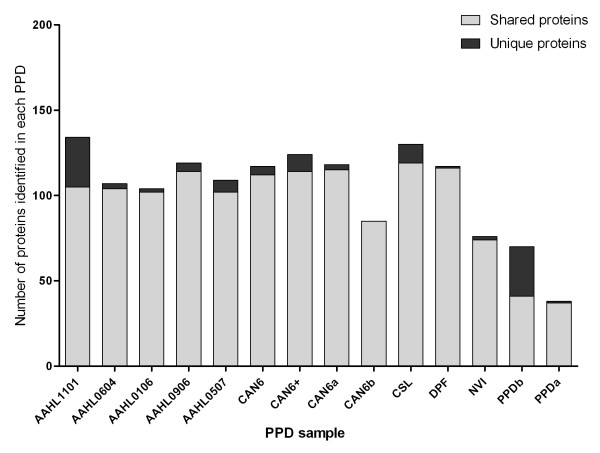
**The number of proteins identified by LC-MS/MS within each PPD**. Proteins identified in two or more PPD samples are denoted by *light grey *bars and proteins indentified as unique to individual PPD preparations are denoted by *dark grey *bars.

**Figure 3:**
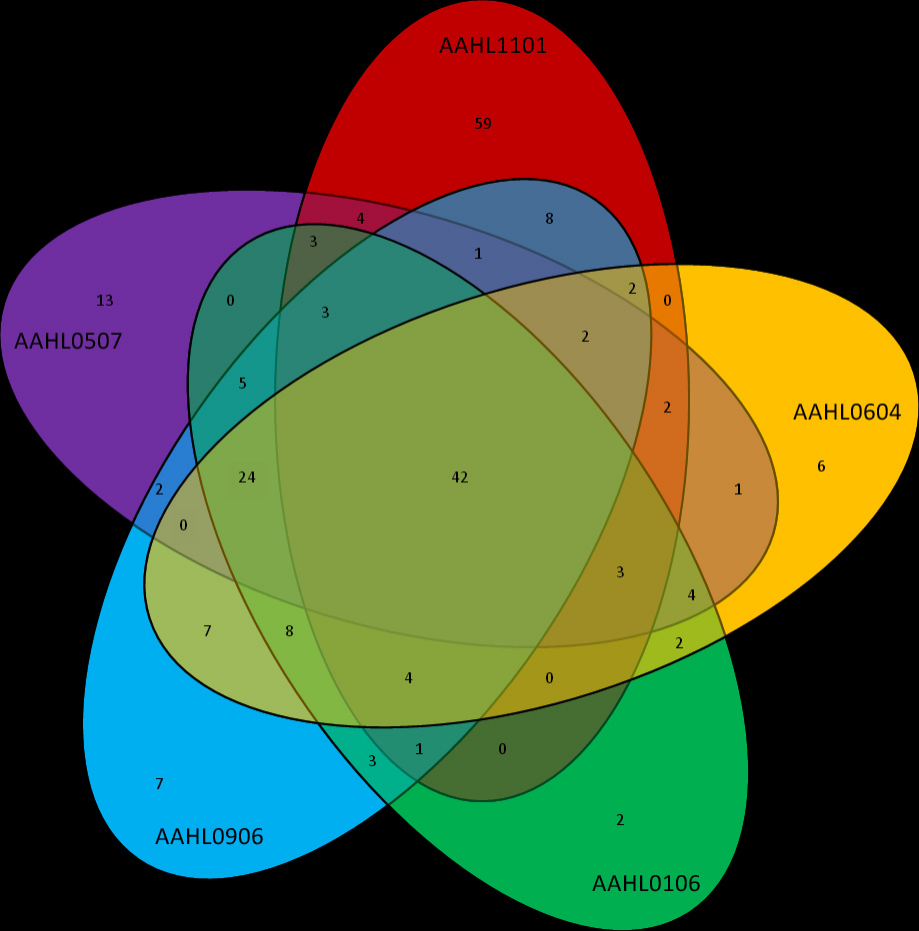
**Five-way Venn diagram of protein composition shared between the five in-house AAHL PPDj preparations**. Numbers within each segment represent the total number of proteins shared by those preparations.

A proteomic comparison by mass spectrometry of all PPD preparations demonstrated significant overlap in composition between preparations (Figure 2). A total of 269 proteins were identified by TripleTOF™ mass spectrometry across all 13 PPDj (Additional file 1: Table S1). The largest number of proteins (134) was identified in the AAHL1101 PPDj sample. The NVSL PPDj sample contained only six detectable proteins. Such a low protein composition seems unlikely given the complexity of other PPDj preparations. Mass spectrometry was repeated on the NVSL PPDj sample using LC-MALDI and a similar number of proteins were identified (data not shown). It was concluded that this PPDj sample has undergone degradation since initial preparation and it was therefore excluded from further analysis. All other PPDj samples contained a similar number of proteins (range 76-134). The majority of these proteins were shared between one or more PPDj preparations (Figure 2). It is interesting to note that while all PPDj preparations contained a small number of unique proteins, the overall composition was conserved across the 12 PPDj samples. Twenty nine proteins were found to be common to all PPDj samples (Table 1). These included many known immunogenic antigens, such as the heat shock proteins *GroES*, *GroEL *and *DnaK*. The five AAHL PPDj preparations shared 42 common proteins (Figure 3). The AAHL1101 contained considerably more unique proteins (59) compared to the other four AAHL preparations (Figure 3). Considering that the five AAHL PPDj preparations were produced identically it is surprising that the AAHL1101 preparation contained so many more unique proteins. This finding suggests that despite using a standardised strain and production protocol inherent variation between PPDj preparations will still exist, albeit to a lesser extent than if no standardisation was undertaken.

**Table 1:** Most common proteins observed in 12 PPDj samples

Accession	**Locus_tag**^**‡**^	**Locus_tag**^**¥**^	Gene	Mass (Da)	Product	PPDb	PPDa	20 W sec	34 W sec
P60533	MAPK_4266	MAP_4264	*groES*	10716.1	10 kDa chaperonin	+	+	+	+
Q73YF9	MAPK_1771	MAP_1997	*acpM*	12452.9	acyl carrier protein	+	+	+	+
Q8VU82	MAPK_2159	MAP_1609c	*fbpB*	34692.8	fibronectin-binding antigen 85 complex B	+	+	+	+
Q00488	MAPK_3842	MAP_3840	*dnaK*	66518.5	molecular chaperone DnaK	+	+	+	+
Q50320	MAPK_0241	MAP_3527	*pepA*	35709.1	serine protease	+	+	+	+
Q73UE0	MAPK_0340	MAP_3428c		23791.0	cutinase-related		+	+	+
Q73WQ3	MAPK_1161	MAP_2607c	*fdxC*	11792.1	ferredoxin	+	+	+	+
Q8GF25	MAPK_2115	MAP_1653	*tpx*	16684.8	thiol peroxidase		+	+	+
Q73YR6	MAPK_1879	MAP_1889c	*wag31*	28049.9	secreted antigen Wag31	+	+		+
Q73ZR1	MAPK_2228	MAP_1540	*cfp17*	16715.2	forkhead-domain protein	+	+		+
Q741F3	MAPK_2630	MAP_1138c	*lprG*	24720.8	lipoprotein	+	+		
P42384	MAPK_3938	MAP_3936	*groL2*	56642.6	chaperonin GroEL2	+	+	+	+
Q741G8	MAPK_2646	MAP_1122	*mIHF*	12176.2	integration host factor MihF	+	+	+	+
A0QME6	MAPK_0122	MAP_3646	*pckA*	67659.5	phosphoenolpyruvate carboxykinase (GTP)			+	
P60545	MAPK_4267	MAP_4265	*groL1*	55793.3	chaperonin GroEL	+		+	
P61976	MAPK_1227	MAP_2541c	*mdh*	34599.2	malate dehydrogenase			+	+
Q73TQ9	MAPK_0109	MAP_3659		53750.8	long-chain-fatty-acid--CoA ligase				+
Q73VF3	MAPK_0707	MAP_3061c	*fixA*	27847.4	electron transfer flavoprotein (beta-subunit)		+	+	+
Q73VF4	MAPK_0708	MAP_3060c	*fixB*	32085.4	electron transfer flavoprotein (alpha-subunit)	+		+	
Q73X57	MAPK_1315	MAP_2453c	*atpA*	59992.9	H(+)-transporting two-sector ATPase, alpha subunit				+
P61336	MAPK_0813	MAP_2955c	*tsf*	29065.9	elongation factor Tsf	+		+	+
Q73XM7	MAPK_1486	MAP_2282c	*tig*	50618.2	trigger factor				
Q741U7	MAPK_2778	MAP_0990	*eno*	44841.2	phosphopyruvate hydratase				
A0QLN4	MAPK_3950	MAP_3948c		17094.2	hypothetical protein		+	+	+
D5PC72	MAPK_4128	MAP_4126	*rplL*	13430.4	ribosomal protein L7/L12	+	+	+	+
Q73SD1	MAPK_4145	MAP_4143	*tuf*	43738.7	protein-synthesizing GTPase	+	+	+	+
Q73ZL3	MAPK_2179	MAP_1589c	*ahpC*	21641.4	peroxiredoxin subunit C			+	+
A0QCX8	MAPK_1317	MAP_2451c	*atpD*	53141.2	H(+)-transporting two-sector ATPase, beta subunit				
Q73S77	MAPK_4201	MAP_4199	*adk*	19935.7	adenylate kinase	+		+	+

The accession number for the protein with highest sequence coverage is given. Locus tag from the revised K10 genome sequence **^‡ ^**and original K10 genome sequence ^¥^, gene name, mass (Da) and protein name for the MAP K10 ortholog is provided. Proteins also identified in PPDb, PPDa, 20 week or 34 week culture filtrate are indicated by +

**Figure 4:**
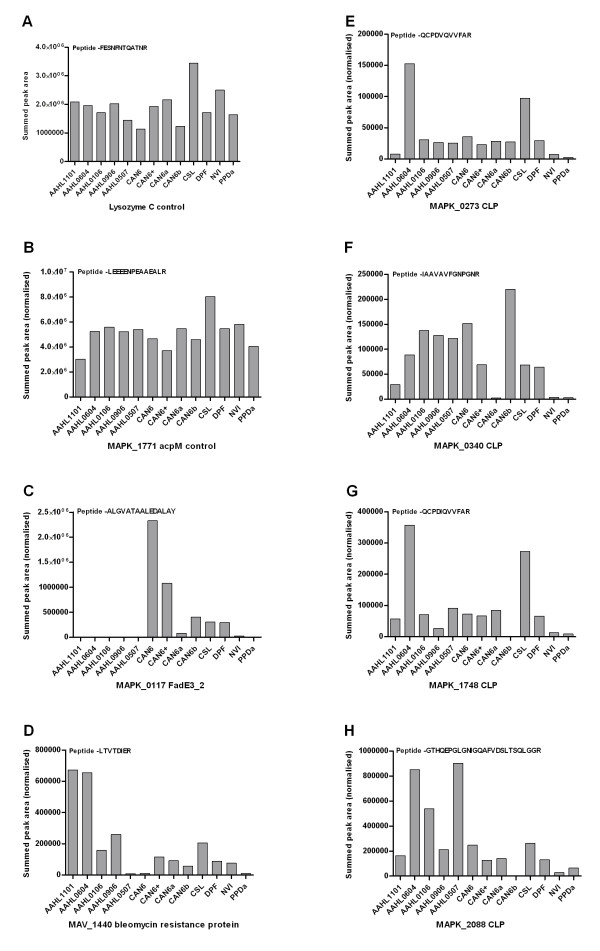
**MRM peptide quantification for lysozyme C control (**A**); MAPK_1771 acyl carrier protein (acpM) control (**B**); MAPK_0117 acyl-CoA dehydrogenase FadE3_2 (**C**); MAV_1440 a glyoxalase/bleomycin resistance protein (**D**) and four cutinase like proteins (CLP) MAPK_0273, MAPK_0340, MAPK_1748 and MAPK_2088 (E-H)**. The target peptide sequence is also presented in the top right-hand corner of each plot.

The variable nature of the PPDj may be attributed, to some extent, to the different MAP strain type used to produce PPDj globally. Significant genomic differences, including a 7.9 kb deletion, have been observed in certain MAP strains used to prepare PPDj [19]. Furthermore single PPDj preparations maybe produced from combining a number of bacterial cultures of different strains, thus increasing the inherent variability. In our laboratory a single wild-type bovine isolate (CLIJ623 [23]) was used to produce PPDj. We have recently found that this Australian MAP isolate has distinct genetic differences compared with the US reference isolate K10 [4,26]. Many of these genetic polymorphisms translated into protein variation which may be reflected in differences in PPDj composition. The absence of the acyl-CoA dehydrogenase FadE3_2 (MAPK_0117) from the AAHL PPDj preparations is a perfect example of genetic strain differences affecting proteomic composition of PPDj. Multiple reaction monitoring (MRM) analysis was used to qualify the relative abundance of FadE3_2 across different PPDj and PPDa samples. Based on a single C-terminal peptide, MRM demonstrated a complete absence of this peptide in all AAHL PPD preparations (Figure 4C). Our previous genome sequencing studies have shown that this gene is truncated by 149 amino acids in the CLIJ623 genome [4]. This truncation was a consequence of a single base frame shift deletion which was also observed in MAP isolates derived from humans. Conversely, K10, and other bovine MAP strains sequenced in our laboratory, appeared to retain an intact copy of this gene. It's also possible that the truncated form of this protein is still expressed in the MAP strains which contain this SNP. However additional experimentation would be required to confirm this. Regardless this situation clearly demonstrates the effects of strain differences on PPDj composition and highlights the need for a well characterised reference strain for all PPDj production globally. Ideally such a reference strain would contain as many antigenic open reading frames as possible.

One of the most common families of proteins identified across all PPDj preparations was the cutinase-like proteins (CLPs). Cutinases are serine hydrolases which cleave carboxylic ester bonds of glycolipid polymers thus producing the cutin monomer. The immunogenicity of cutinase enzymes have been demonstrated previously [27]. *M. tuberculosis *contains seven CLPs [27] and four of these were found in tuberculin PPD [16]. We identified seven different CLPs in PPDj (Additional file 1: Table S1) with the most common CLPs arising from locus tags MAPK_0273, MAPK_0340, MAPK_1748 and MAPK_2088. MRM quantification of single peptides corresponding to these CLPs revealed significant differences in the absolute abundance between PPDj and PPDa preparations (Figure 4E-3H). This finding suggests that, while the protein composition of different PPDj preparations is similar in terms of presence or absence of proteins, the abundance of individual components varies significantly. Both MAPK_1748 and MAPK_2088 were in fact absent from the CAN6b PPDj preparation. This may suggest that these CLPs are less important for antigenic specificity compared to the other CLPs. Interestingly the seven CLPs identified in PPDj appear to be conserved in the *Mycobacterium avium *complex with orthologs in *M. bovis *more divergent based on amino acid sequence (Table 2). Specific CLPs have been shown to elicit strong IFN-γ responses, and when used as vaccine candidates, they conferred a moderate protection to *M. tuberculosis *in a murine model [27]. Furthermore, the IFN-γ response was specific to individual CLPs and demonstrated limited cross reactivity [27]. At least two CLPs were also detected in PPDa in the study of Borsuk et al. [16], however, these proteins were significantly divergent to those identified in PPDj. MRM analysis demonstrated that while CLPs are present in PPDa they are significantly less abundant compared to most PPDj preparations. Considering the diverse nature of CLPs across the *Mycobacterium *genus, as well as their immunogenic ability to elicit a strong IFN-γ response, these enzymes should be considered as possible diagnostic antigens to replace PPDj.

**Table 2:** Similarity of amino acid sequence of cutinase-like proteins from MAP

Locus Tags	**Similarity to Mb**^**†**^	**Similarity to MAH**^**±**^	**Similarity to MAA**^**γ**^
^**‡**^MAPK_0273/^**¥**^MAP_3495c	50% to Mb3751	88% to MAV_2169	100% to MaviaA2_23256

^**‡**^MAPK_0340/^**¥**^MAP_3428c	46% to Mb3482	99% to MAV_4283	99% to MaviaA2_18901

^**‡**^MAPK_1464/^**¥**^MAP_2304	51% to Mb2006c	99% to MAV_1682	99% to MaviaA2_010100007523

^**‡**^MAPK_1748/^**¥**^MAP_2020	55% to Mb1788	96% to MAV_2169	100% to MaviaA2_010100009430

^**‡**^MAPK_2088/^**¥**^MAP_1680c	75% to Mb2006c	98% to MAV_2741	99% to MaviaA2_11201

^**‡**^MAPK_3435/^**¥**^MAP_0333	78% to Mb3751	100% to MAV_0369	99% to MaviaA2_01656

^**‡**^MAPK_4239/^**¥**^MAP_4237c	74% to Mb3481	99% to MAV_4394	99% to MaviaA2_010100019466

Compared to *Mycobacterium bovis***^†^**, *Mycobacterium avium *subsp. *hominissuis *^± ^and *Mycobacterium avium *subsp. *avium*^γ^. The closest ortholog locus tag is also indicated. The MAP Locus tags from the revised K10 genome sequence ^‡ ^and original K10 genome sequence ^¥ ^are indicated

Decreasing the inherent cross reactivity against non-MAP mycobacterial antigens remains a significant challenge for improving JD diagnosis. In an attempt to identify proteins which are unique to PPDj, we also characterised the proteomic composition of PPD preparations produced from *Mycobacterium avium *subsp. *avium *(PPDa) and *Mycobacterium bovis *(PPDb). These PPD preparations are commonly used as control antigens in IFN-γ tests to control for antigenic cross reactivity against environmental non-MAP mycobacteria. Interestingly, PPDa contained significantly less proteins compared to PPDj and PPDb (Figure 2). This finding is in agreement with a study by Santema et al. [17] who demonstrated that the composition of PPDa was significantly less complex compared with PPDj preparations. Nevertheless our study investigated only one PPDa preparation and additional preparations should be examined to confirm this finding. Despite fewer proteins, PPDa was highly similar in composition to PPDj. Interestingly, many of the most common components of PPDj were also identified in PPDa and PPDb (Table 1). MRM analysis demonstrated that PPDa had very similar abundance of the acyl carrier protein *acpM *compared to PPDj (Figure 4B). Conserved proteins that are shared between different mycobacterial PPD preparations undoubtedly increase the likelihood of cross reactivity. Two proteins that were found to be unique to PPDj are the peroxiredoxin subunits C and D (*ahpC *and *ahpD*). These proteins have been evaluated as potential diagnostic antigens previously and have been shown to be specific to MAP with little cross reactivity against other mycobacteria [28,29]. A recent review by Mikkelsen et al. [30] suggests *ahpC *and *ahpD *are two of the most promising antigens for measuring specific cell mediated immunity. Other proteins which were common in PPDj, but absent in PPDa and PPDb include phosphoenolpyruvate carboxykinase (*pckA*), malate dehydrogenase (*mdh*), the molecular chaperone trigger factor (*tig*) and phosphopyruvate hydratase (*eno*). The immunogenicity of these proteins, however, remains to be evaluated.

In an attempt to identify the optimal harvest time point for PPDj preparation temporal differences in protein secretion of *in vitro *cultured MAP (CLIJ623) was examined. The number of secreted proteins identified in the cell free culture filtrates increased with age from 72 individual proteins at 20 weeks to 97 proteins at 34 weeks (Additional file 2: Table S2). Interestingly, many proteins identified in the cell-free culture filtrate did not contain a signal peptide and were thus not expected to be secreted. In fact 74% of proteins identified in the cell-free culture filtrate had a signal peptide probability of < 0.5. This finding suggests intracellular proteins are frequently released following cell death and autolysis at these time points. We hypothesised that the secretome is comparable to PPDj, in that PPDj should contain all secretome proteins, as well as some cellular proteins. However, while many of the secreted proteins were also found in PPDj, approximately 20% of these proteins were not observed in PPDj. Furthermore, only 44 proteins were secreted at both time points. This result suggests that PPDj composition may vary significantly depending on the age of the culture. Previously, we have demonstrated that many of the major components of PPDj, such as GroEL2 (*hsp65*), bacterioferritin and *ahpC*, are not secreted in large quantities in *Mycobacterium avium *subsp. *avium *at either 7 or 14 weeks [31]. In our laboratory *Mycobacterium avium *subsp. *avium *PPDa is produced from 13 week cultures and thus may have vast quantitative differences in protein composition compared to PPDj which is produced from 24 week cultures.

Database interrogation revealed that some proteins identified in PPDj had higher sequence coverage to other *Mycobacterium *species compared to the MAP K10 reference. This situation most likely stems from either incorrectly annotated start open reading frames in MAP K10 or simply that the open reading frame has not been annotated onto the K10 genome at all. Our study identified at least five cases of the latter (Additional file 3: Table S3). Homology searches on the K10 genome revealed six un-annotated open reading frames corresponding to these five proteins. One of these proteins was a glyoxalase/bleomycin resistance protein, closely resembling MAV_1440 in *Mycobacterium avium *(strain 104). Interestingly, initial mass spectrometry analysis showed this protein to be unique to AAHL PPDj preparations (Additional file 1: Table S1) however, MRM analysis demonstrates that while it is generally more abundant in AAHL PPDj samples, it could also be detected in other PPDj and PPDa preparations (Figure 4D).

## Conclusions

This study describes the production and proteomic characterisation of PPDj within our laboratory. The proteomic compositions of the PPDj preparations produced in our laboratory are highly similar to PPDj preparations produced in other institutes/laboratories in terms of general composition. However, considerable differences in absolute abundance of specific proteins were also observed. Compositional differences between PPDj preparations are to some extent a direct reflection of genetic differences between the MAP strain types and indirect temporal changes in growth stages and harvest time point. We strongly advocate the use of a well characterised MAP reference strain, with well described growth dynamics, for all PPDj production globally. Furthermore a standardised harvest time point at 24 weeks will reduce the influence of temporal changes in protein expression thereby generating a more uniform preparation. The immunogenic CLPs identified in across the PPDj preparations should be further evaluated as possible diagnostic targets.

## Methods

### MAP inoculum

All PPDs produced within our laboratory were derived from an Australian wild type bovine MAP isolate referred to as CLIJ623. This isolate was recovered from the ileocaecal valve of a Jersey cow exhibiting clinical symptoms of the terminal stages of Johne's disease. It was typed as a cattle (Type II) strain by a number of tests: typical colony formation on Herrold's Egg Yolk medium (HEYM) supplemented with mycobactin and sodium pyruvate [32-34], mycobactin dependency, characteristic acid-fast microscopic morphology, PCR detection of the insertion element IS*900 *and restriction enzyme analysis of IS*1311 *[23,35-39]. This isolate has also recently undergone whole genome sequencing [4]. This virulent Australian wild-type strain, with minimal laboratory passage (less than 3 passages), has been used in long term infection time course experiments in cattle, sheep and goats [23-25].

### Preparation and source of PPD

Five batches of PPDj were produced between 2001 and 2007 within our laboratory. All five PPDj preparations were produced identically using the same MAP strain and preparation procedure and are considered biological replicates. PPDj was prepared using a modified protocol originally provided by CSL Limited. The mother seed of CLIJ623 was first grown on HEYM slopes supplemented with 2 mg/L of Mycobactin J (Allied Monitor Inc, Fayette, USA). and sodium pyruvate for eight to twelve weeks. HEYM slopes were prepared exactly as described in the Australia and New Zealand Standard Diagnostic Procedures (ANZSDPs) for Paratuberculosis http://www.scahls.org.au/procedures/anzsdps. Approximately 10^8 ^bacilli per mL were then inoculated into modified Watson and Reid media (media volume to flask volume 1/10). The modified Watson and Reid media was prepared as previously described by Morrison [40] and contained 2 mg/L of Mycobactin J (Allied Monitor Inc). The final pH was adjusted to between 5.8-5.9 using NaOH. Growth was monitored weekly during a stationary incubation for 24 weeks after which time a thick dominant pellicle was observed. At this point cultures were inactivated in a steam autoclave at 100°C for 2 h and cooled to 4°C overnight. Sterilised cultures were filtered through sterile gauze and transferred into sterile centrifugation pots for centrifugation at 3,000 × *g *for 10 min. The resultant supernatants were transferred to new pre-weighed centrifuge pots and 40% (w/v) TCA solution in distilled water was added to final concentration of 4%. The mixture was stirred for at least 30 min with magnetic stirrer and placed (without stirring) in the dark room at room temperature for 15-18 h. During this period the tuberculo-proteins were precipitated by the TCA. Following precipitation the preparation was centrifuged at 2,600 × *g *for 15 min. This step generated a soft pellet that was often difficult to retain and a second centrifugation step was sometimes required. Once the pellet was well formed the supernatant was carefully decanted and pellets retained. The resulting pellet was washed with 5% NaCl (w/v) + 0.5% phenol (w/v) (adjusted to pH 3.0 with 40% (w/v) TCA). Pots were centrifuged at 2,600 × *g *for 15 min and washing was repeated three times until the pH of the supernatant was between 2 and 3, and the amount of wet-weight was measured. The washed pellets were dissolved in 1.8% (w/v) Na_2_HPO_4_. 2H_2_O (pH 11) with the amount added equalling approximately 2-2.5 mL per gram of wet-weight. Each protein pellet dissolved completely within 90-120 min and the pH of the resultant preparation was 9.7. These preparations were centrifuged at 2,600 × *g *for 10 min and supernatants were mixed with an equal volume of phosphate buffer (1.5% (w/v) KH_2_PO_4 _+ 3% (w/v) Na_2_HPO_4_. 2H_2_O) containing 19.4% (w/v) glucose and 0.5% (w/v) phenol. The pH of each preparation was checked to be in the range of 6.7-6.9. Concentrated PPDj preparations were stored at 4°C in the dark. Additional PPD preparations were obtained from various institutes/laboratories as outlines in Table 3. Total protein was quantified for each PPD using the 2-D Quant Kit (GE Healthcare) as per the manufacturer's recommendation.

**Table 3:** List of 15 PPD samples analysed in this study

PPD	Species	Lot	Country	Provider
AAHL1101	MAP	NA	Australia	NA
AAHL0604	MAP	NA	Australia	NA
AAHL0106	MAP	NA	Australia	NA
AAHL0906	MAP	NA	Australia	NA
AAHL0507	MAP	NA	Australia	NA
CSL	MAP	0404-21601	Australia	CSL Limited
CAN 6	MAP	0404-41101:8	Canada	CSL Limited
CAN 6a	MAP	90-00-1:68	Canada	CSL Limited
CAN 6b	MAP	90-00A	Canada	Canadian Food Inspection Agency
CAN 6+	MAP	0404-41101:8	Canada	CSL Limited
DPF	MAP	0404-41101:8	The Netherlands	Pfizer Animal Health
NVI	MAP	NA	Norway	National Veterinary Institute
NVSL	MAP	9801	USA	National Veterinary Service Laboratory (USDA)
PPDa	MAA	2091-02301	Australia	CSL Limited
PPDb	Mb	209000901	Australia	CSL Limited

All PPD samples prefixed with *AAHL *were produced in-house. The NVSL PPDj was excluded from further analysis due to an unexpectedly low number of identified proteins. For species the abbreviation MAP = *Mycobacterium avium *subsp *paratuberculosis*, MAA = *Mycobacterium avium *subsp. *avium*, and Mb = *Mycobacterium bovis*.

### IFN-γ assay

The in-house PPDj preparation, AAHL1101, was compared to the PPDj preparations produced in Canadian (CAN6) and CSL in its ability to stimulate the IFN-γ response in both Holstein-Friesian cattle and Merino sheep experimentally infected with JD. Blood sample for IFN-γ testing were obtained from previous animal infection experiments described by Stewart et al [23,25]. Briefly, Holstein-Friesian calves (six weeks of age) or Merino sheep (six months of age) were experimentally infected at weekly intervals for four weeks with either 1 × 10^10^-2 × 10^10 ^cultured bacteria (bovine MAP strain) or JD infected ileal and distal jejunal mucosal scaping. Previous studies have demonstrated that infection is more easily established with an infected mucosal inoculum compared to a pure bacterial challenge [23-25]. The control groups were dosed with Watson and Reid media without mycobactin. For the purpose of comparing PPDj preparations blood samples taken 45 months post challenge were used. Neither cattle nor sheep demonstrated clinical signs of disease (faecal shedding, loss of body weight and diarrhoea) at this time point. However, positive faecal culture - indicative of a subclinical infection - was observed for both infected cattle and sheep previous to this time point [23,25].

The IFN-γ assay was performed on duplicate plasma samples with BOVIGAM™ kits following stimulation of blood with either sterile phosphate-buffered saline (nil antigen), *Mycobacterium avium *PPDa or one of three PPDj samples. The IFN-γ assay was performed exactly as previously described [23] using whole blood samples obtained from either Holstein-Friesian cattle or Merino sheep experimentally infected with MAP (through either tissue or bacterial inoculum) as described by Stewart et al. [23,25]. The assays were performed on single plates and the results are reported as optical densities. The response of infected animals to AAHL1101, CSL or CAN6 PPDj was compared using a One-way analysis of variance (ANOVA).

### Time course secretome

The composition of secreted MAP proteins within the culture media was investigated at two independent time points. Temporal differences in protein secretion of *in vitro *cultured MAP may significant influence PPDj composition. The bovine strain CLIJ623 was cultured in modified Watson and Reid (protein free) media as described above. Culture media was harvested at 20 and 34 weeks post inoculation. Media was concentrated using an Amicon filter membrane as previously described [31]. Total protein was quantified for each PPD using the 2-D Quant Kit (GE Healthcare) as per the manufacturer's recommendation. Fifty micrograms of protein was then analysed by LC-MS/MS as described below.

### Trypsin digest

A total of 15 PPD and two secretome samples were analysed by mass spectrometry (Table 3). This included five PPDj preparations prepared within our laboratory and ten additional PPD samples obtained from other institutes/laboratories. These additional PPD preparations were derived from Australia, Canada, The Netherlands, Norway and the USA and were derived from *Mycobacterium avium *subsp. *paratuberculosis *(PPDj), *Mycobacterium avium *subsp. *avium *(PPDa) and *Mycobacterium bovis *(PPDb). The PPD preparations (50 μg) were first subjected to reduction with 0.2 mM dithiothreitol (DTT) in 40 mM NH_4_HCO_3 _for 2 h at 37°C, followed by alkylation with 50 mM iodoacetamide for 20 mins in the dark at room temperature. PPD proteins were then digested overnight with 2.5 μg of trypsin (protein sequencing grade; Sigma Aldrich) at 37°C. Digestion was terminated by the addition of 0.8% (w/v) formic acid and PPD digests were dried by vacuum centrifugation.

### Chromatography

Protein digests were reconstituted in 0.1% formic acid and 1 μg of tryptic peptides were chromatographically resolved using a Shimadzu Prominence LC20 HPLC system with a C18 Vydac column (75 μm × 15 cm, 300 Å, 5 μm). A linear gradient at flow rate of 800 nL/min from 1-40% solvent B over 80 min was utilised where solvent A was 0.1% (w/v) formic acid and solvent B was 0.1% (w/v) formic acid in 90% acetonitrile.

### Mass Spectrometry

The eluate from the HPLC system was directly coupled to the nanoelectrospray ionisation source of a TripleTOF™ 5600 system (AB/Sciex, Foster City, CA, USA). Data were acquired in information dependent acquisition (IDA) mode. The IDA method consisted of a high resolution TOF-MS survey scan followed by 20 MS/MS in a second with a maximum accumulation time of 50 ms. First stage MS analysis was performed in positive ion mode over the mass range *m/z *300-2000 with a 0.5 s accumulation time. The ionspray voltage was set to 2600 V, the curtain gas was set to 25, the nebuliser gas to 20 and the heated interface was set to 150°C. Tandem mass spectra were acquired over the mass range *m/z *100-2000 using rolling collision energy (CE) for optimum peptide fragmentation. Precursor ion masses were excluded for 8 s after two occurrences.

### Database searching and false discovery rate analysis

All data were processed using ProteinPilot v4.0 with integrated false discovery rate analysis. The spectral sets were searched against all Mycobacteriaceae proteins present in the Uniprot database (version 20110721; 430,740 proteins). Search parameters were defined as cysteine alkylation with iodoacetamide, trypsin as the digestion enzyme and no restrictions were placed on taxonomy. Modifications were set to the "generic workup" and "biological" modification sets provided with this software package, which consisted of all modifications listed in Unimod, for example, acetylation, methylation and phosphorylation. The generic workup modifications set contains 59 potential modifications that may occur as a result of sample handling, for example, oxidation, dehydration and deamidation. The identification of proteins was recorded in the Results section if MS/MS spectral scores were achieved at the *p *< 0.01 confidence level, i.e. at a 1% global false discovery rate (FDR). Only proteins for which two peptides were identified were considered to be present. Signal peptide probability was determined using SignalP 3.0 [41].

### Multiple reaction monitoring (MRM)

Quantification of individual proteins was compared between PPDj and PPDa preparations based on MRM mass spectrometry analysis. A total of 50 μg of lysozyme C (Sigma-Aldrich) was trypsin digested as described above. Each PPDj and PPDa sample was then spiked with 0.05 μg of digested lysozyme C and analysed on a 4000 QTRAP mass spectrometer (Applied Biosystems, Framingham, MA, USA) equipped with a TurboV ionization source operated in positive ion mode. Samples were chromatographically separated on a Shimadzu Prominence LC20 HPLC system with a C18 Vydac column (75 μm × 15 cm, 300 Å, 5 μm). A linear gradient at flowrate of 800 nL/min from 1-40% solvent B over 20 min was utilised where solvent A was 0.1% formic acid and solvent B was 0.1% (w/v) formic acid in 90% acetonitrile. The eluent from the HPLC was directly coupled to the mass spectrometer. Data were acquired and processed using Analyst 1.5 software™ Quantification of MAP peptides was achieved using scheduled MRM scanning experiments using a 120 s detection window for each MRM transition and a 1 s cycle time. A total of seven target proteins were quantified including four cutinase-like proteins (Locus tags; MAPK_0340, MAPK_0273, MAPK_1748 and MAPK_2088), the acyl-CoA dehydrogenase FadE3_2 (MAPK_0117), a glyoxalase/bleomycin resistance protein (MAV_1440) and the acyl carrier protein acpM (MAPK_1771). For each protein a single peptide was chosen for quantification using the summed area of the two most intense MRM transitions. MRM peak areas were normalised using the MRM peak area of the positive control peptides from lysozyme C (Figure 4A).

## Abbreviations

CLP: Cutinase-like proteins; CMI: Cell mediated immunity; DTT: Dithiothreitol; ELISA: Enzyme-linked immunosorbent assay; HEYM: Herrold's Egg Yolk medium; JD: Johne's disease; LC-MALDI: Liquid chromatography matrix-assisted laser desorption/ionization; LC-MS/MS: Liquid chromatography tandem mass spectrometry; MAA: *Mycobacterium avium *subspecies *avium*; MAH: *Mycobacterium avium *subspecies *hominissuis*; MAP: *Mycobacterium avium *subspecies *paratuberculosis*; Mb: *Mycobacterium bovis*; IFN-γ: Gamma interferon; PPD: purified protein derivative; TCA: trichloroacetic acid.

## Competing interests

The authors declare that they have no competing interests.

## Authors' contributions

JV and GB prepared PPDj. BS and MC conducted mass spectrometry experiments. JW participated in experimental design, conducted data analysis, bioinformatics and drafted the manuscript. WM conceived the study and participated in experimental design. All authors read, contributed to and approved the final manuscript.

## Supplementary Material

Additional file 1 **Table S1**. Proteins identified in each PPD are indicated by +. UniProt accession number for the protein with highest sequence coverage is given. K10 locus tag (revised version [26] and original version [42]), gene name, mass (Da) and protein name for the MAP K10 ortholog is provided. Ortholog locus tags for proteins identified in PPDa and PPDb which were also found in PPDj are provided. Click here for file

Additional file 2 **Table S2**. Proteins identified in culture filtrate at 20 and 34 weeks post inoculation are indicated by +. UniProt accession number for the protein with highest sequence coverage is given. K10 locus tag (revised version [26] and original version [42]), gene name, mass (Da) and protein name for the MAP K10 ortholog is provided. Proteins that were also identified in one or more PPDj are indicated by +. Signal peptide probability was calculated by SignalP 3.0. Click here for file

Additional file 3 **Table S3**. Description of non-annotated proteins identified in PPDj. The identified *M. avium *104 ortholog locus tag is provided, along with genome coordinated where the open reading frames are positioned in the K10 genome. Click here for file
